# Cardiovascular diseases in Quebec health administrative databases: missing diagnoses and underestimation of the number of cases in a 28-year prospective cohort

**DOI:** 10.24095/hpcdp.44.10.01

**Published:** 2024-10

**Authors:** Mathilde Lavigne-Robichaud, Edwige Tiwa Diffo, Chantal Brisson, Manon Levesque, Caty Blanchette, Alain Milot, Denis Talbot, Xavier Trudel

**Affiliations:** 1 Faculty of Medicine, Universit Laval, Qubec City, Quebec, Canada; 2 Centre de recherche du CHU de Qubec—Universit Laval, Qubec City, Quebec, Canada

**Keywords:** cardiovascular disease, surveillance, epidemiology, cohort study, missing diagnoses, coronary heart disease, stroke, Canada

## Abstract

**Introduction:**

Cardiovascular disease (CVD) surveillance in Quebec and the rest of Canada is carried out using health administrative databases, which in Quebec includes the physician claims database. The presence of billing claims without diagnoses can lead to the number of CVD cases being underestimated. The purpose of this study is to estimate the proportion of CVD diagnoses and CVD cases that may be missing from these databases.

**Methods:**

The study was conducted using a prospective cohort of 8781participants living in the Qubec City area. Access to health administrative databases was granted for the entire 28-year follow-up period. First, we performed frequency analyses to estimate the proportion of missing CVD diagnoses. Then we used validated algorithms to identify CVD cases and estimate the proportion of CVD cases that were potentially not captured over the 28-year period.

**Results:**

About one-fifth (22.1%) of the diagnoses in the physician claims database were missing. The proportion of missing CVD cases was estimated at 12.7% for 1991–2018, although this varied with the period covered (1991–1996: 15.5%; 1997–2013: 10.7%; and 2014–2018: 16.3%).

**Conclusion:**

Approximatively 1 in 10 CVD cases are not identified due to a missing diagnosis. This underestimation of CVD cases is a potential limitation that should be considered when using Quebec health administrative databases to identify CVD cases for surveillance work and epidemiological studies.

**Highlights**

- Health administrative databases are an important source of data on cardiovascular diseases (CVDs) in Quebec and elsewhere in Canada.
- We estimated the number of CVD cases that are potentially missed as a result of diagnoses not captured in Quebec health administrative databases.
- Using the health administrative data of 8781 participants who were followed for 28 years, we estimated that 12.7% of CVD cases may be overlooked because of missing diagnoses.
- Underestimating CVD cases is a limitation that should be considered in surveillance work and epidemiological studies that use health administrative databases.

## Introduction

Cardiovascular diseases (CVDs) are the leading cause of death worldwide and the second most common cause of death in Canada, after cancer.^1^,^2^ Approximately 2.4million Canadians live with heart disease, which is the leading cause of years of life lost.^3^ The economic burden of CVDs is significant because of the associated direct and indirect costs.^4^

Valid estimates of the prevalence and incidence of CVDs are required for monitoring and research purposes. Such estimates are also necessary for supporting the development and evaluation of the effectiveness of preventive strategies aimed at reducing the burden of CVDs in the population.^5^,^6^ In a universal health care system like the one in Canada, health administrative databases are a valuable source of data on health conditions because they are accessible and inexpensive and provide near-universal coverage of the population over extensive periods of time.

The Quebec Integrated Chronic Disease Surveillance System (QICDSS) conducts CVD surveillance in Quebec. The prevalence and incidence of CVDs are identified using health administrative databases, including the hospitalization database (MED-CHO), the physician claims database and the vital statistics death database.^7^In order to derive a valid estimate of the prevalence and incidence of CVDs, it is vital that these health administrative databases contain high quality data. The completeness of diagnoses is an essential element of the quality of such data;^8^ a high proportion of missing data can undermine both the validity and accuracy of resulting estimates.

In Quebec, the completeness of diagnoses varies with the health administrative database.^8^ The completeness of diagnoses was 100% in hospital records and between 88% and 93%, depending on the type of services, in physician claims databases.8 Wilchesky et al.^9^ found that approximately 30% of physician billing claims had missing or invalid diagnoses in the physician claims database. Although these studies suggest that a large proportion of diagnoses are missing in the physician claims database, no studies have documented the proportion of diagnoses missing for CVD nor the potential underestimation of CVD cases resulting from missing diagnoses.

The objective of this study was to estimate the proportion of missing diagnoses for CVD in the Quebec health administrative databases as well as the resulting impact on the number of identified CVD cases in a prospective cohort of 8781 women and men followed for 28 years. Specifically, we estimated the proportion of missing diagnoses that are potential diagnoses of CVD and the impact of these missing diagnoses on the estimate of the number of CVD cases.

## Methods

***Ethics approval***

This study was approved by the ethics committee of the Centre de recherche du CHU de Qubec—Universit Laval (reference number 12-1674).

***Description of cohort***

The PROspective Qubec (PROQ) Study on Work and Health is a prospective cohort that initially comprised 9188 white-collar workers (49.9% female and 50.1% male) from 19 public and quasi-public organizations in the Qubec City area.^10^ Average age was 40.2 years. Cohort participants were employed in management (10.4%), professional/teaching (34.0%) and technical/other (55.6%) positions. Education levels varied: 42.1% of participants had a university degree, 28.1% had a college diploma and 29.8% had neither. Annual family income at recruitment ranged from less than $30000 (14.9%) to $70000 or more (28.0%).

***Population***

At recruitment, the participation rate was 75%. Of the initial cohort (n = 9188), 380 refused access to their health administrative data, 14 gave their consent but could not be matched to the Rgie de l’assurance maladie du Qubec (RAMQ; the health insurance board of the province of Quebec) and 13 had missing nominative data, preventing matching to RAMQ; the 8781 participants thus represented 96% of the initial cohort. Access to health administrative data was secured for the 28 years of follow-up, until 31 December 2018. 

All participants gave their informed written consent.

***Extraction from health administrative databases***

Diagnostic codes from the *International Classification of Diseases, Ninth Revision* (ICD-9) and *International Statistical Classification of Diseases and Related Health Problems, Tenth Revision* (ICD-10) and intervention codes from the *Canadian Classification of Diagnostic, Therapeutic, and Surgical Procedures* (CCP) and the *Canadian Classification of Health Interventions* (CCI) were extracted from the Quebec physician claims database and the Quebec hospitalization database (MED-CHO) for the 1991–2018 period. The dates, medical procedure codes, service locations and types of services associated with these codes were also extracted from these databases.

***Definition of CVD cases***

CVDs considered in this study were coronary heart disease and stroke.^11^,^12^ Participants with myocardial infarction, angina pectoris, and acute and chronic coronary syndromes or who had undergone percutaneous coronary intervention, coronary bypass or angioplasty were identified using the following codes: ICD-9 410–414; ICD-10 I20–I25; CCP 48.02, 48.03, 48.09 and 48.1; and CCI 1.IJ.50, 1.IJ.57.GQ and 1.IJ.76.^13^ Strokes, including transient ischemic attacks, were identified with ICD-9 codes 362.3, 430–432 and 434–436 and with ICD-10 codes I60, I61, I63.X (except I63.6), I64, H34.0, H34.2 and G45.X (except G45.4).^14^

Participants were classified as CVD cases if health administrative records indicated a hospitalization with a diagnosis or procedural code for coronary heart disease or stroke; two medical claims with a diagnosis of coronary heart disease or stroke in the physician claims database within a one-year period; or coronary heart disease or stroke as the primary cause of death in the death records. 

The event occurrence date was defined as the first date when one of these three criteria was met.

These algorithms are used for case definitions by the Institut national de sant publique du Qubec (INSPQ).^15^ Similar algorithms, except for death records, are used by the Canadian Chronic Disease Surveillance System (CCDSS).^3^ These algorithms have been validated in Ontario for coronary heart diseases (sensitivity: 77.0%; specificity: 97.5%)^13^ and stroke (sensitivity: 60.2%; specificity: 99.2%).^14^

***Missing diagnoses***

Only the physician claims database had missing diagnoses. In this database, the breakdown of missing diagnoses, identified by the codes “V999” and “0000” or a blank entry, was examined according to type of services, year and patient characteristics, namely sex, age group (<35years, 35–44 years, ≥45 years) and occupational category (management, professional/teaching and technical/other). Based on the overall trends observed over the entire follow-up period, the periods for which the proportion of missing diagnoses varied were identified (1991–1996, 1997–2013 and 2014–2018). 

**Figure 1:**
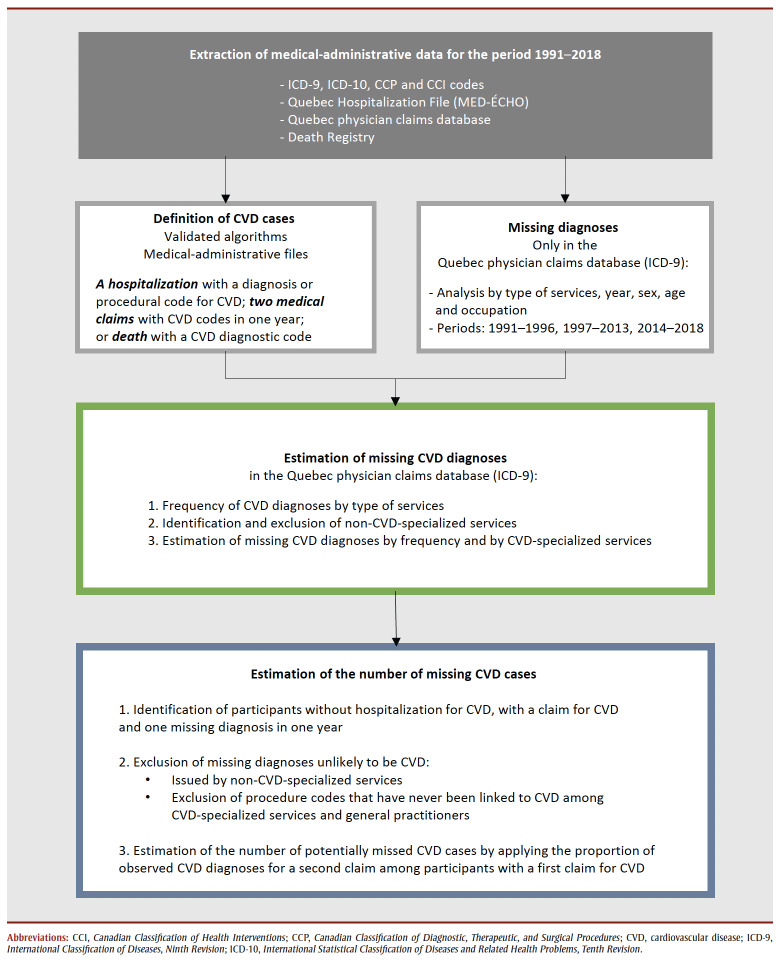
Flow chart showing the process for estimating CVD diagnoses missing from health administrative databases and resulting missing cases

Figure 1 shows a flow chart of the process of estimating CVD diagnoses and cases missing from health administrative databases for the period 1991 to 2018.

***Estimating missing CVD diagnoses***

First, CVD diagnoses were identified using ICD-9 codes (362.3, 410–414,430–432, 434–436), the only classification used in the physician claims database. We then calculated the frequency of types of services associated with those CVD diagnoses. The types of services with at least 18CVD diagnosis codes were classified as “CVD-specialized services.” This threshold of 18 CVD diagnosis codes (for cardiology, diagnostic radiology or ultrasonography, neurology, internal medicine, cardiovascular and thoracic surgery, nuclear medicine, neurosurgery, physical medicine and rehabilitation, anesthesiology, emergency medicine, ophthalmology, general surgery, geriatrics, nephrology, respirology and thoracic surgery) was used because it encompassed 99.9% of all CVD diagnoses, that is, almost complete coverage. Other types of services (e.g. medical microbiology and infectious diseases, diagnostic radiology, hematology, orthopedic surgery, psychiatry, plastic surgery, otolaryngology / neck and face surgery, and public health or occupational preventive medicine) presented very few or no CVD diagnoses and were classified as “non-CVD-specialized services.” Missing diagnoses from these types of services were not included in this analysis given the low probability of these being CVD diagnoses.

We estimated the proportion of missing diagnoses that are potential CVD diagnoses by applying the percentages obtained for CVD diagnoses to the missing diagnoses. Specifically, for each combination of CVD-specialized services and medical procedure codes for a given period, we used frequency analysis to calculate the proportion of the total number of diagnoses that were CVD diagnoses. The resulting percentages were multiplied by the number of associated missing diagnoses to obtain an estimate of the number of potential CVD diagnoses among the missing diagnoses for each combination. Lastly, the number of potential CVD diagnoses were summed to obtain an estimate for the entire period.

***Estimating the number of CVD cases***

We estimated the number of potential CVD cases associated with missing diagnoses using an individual-based approach, according to validated algorithms.

We determined the number of participants without a CVD event (and therefore not hospitalized for CVD) who had a single claim for CVD and at least one other claim with a missing diagnosis within 365 days before or after the claim for CVD. Missing diagnoses with a low probability of being CVD diagnoses were excluded. Thus, all missing diagnoses associated with non-CVD-specialized services were excluded. Likewise, for all CVD-specialized services and general practitioners, we also excluded all missing diagnoses associated with codes for physician claims for which there was no CVD diagnosis during this period and for a given type of service. The resulting estimation is based on the assumption that all remaining missing diagnoses are CVD diagnoses. The estimated proportion of remaining CVD diagnoses was defined as the proportion of second CVD-related claims among participants with a first claim for CVD and a second claim with no missing diagnosis 365days before or after the claim for CVD.

## Results

Of the 8781 participants in our study, 8780 had at least one medical claim recorded in the physician claims database between 1991 and 2018, representing 2 121 856 claims. Only one participant did not have any medical claim during the entire study period. Diagnoses were complete in 77.9% of the claims (1 652 104 /2 121 856). The non-missing diagnoses included 49999 unique claims with a CVD code. Therefore, CVD diagnoses accounted for 3.0% of all the non-missing diagnoses (49 999/1 652 104). The majority (76.4%; 38194/49999) were associated with CVD-specialized services, while general practitioners diagnosed 23.5% (11736/49999); only 0.1% (69/49999) were associated with non-CVD-specialized services. 

About one-fifth of the claims (22.1%; 469752/2121856) presented a missing diagnosis. CVD-specialized services generated 19.9% of these missing diagnoses (93435/469752), while general practitioners generated 27.3% (128391/469752). Thus, the majority (52.8%) of the missing diagnoses were associated with non-CVD-specialized services (Table 1).

**Table 1:** Number and proportion of claims by diagnosis and type of services, Quebec, Canada, 1991–2018

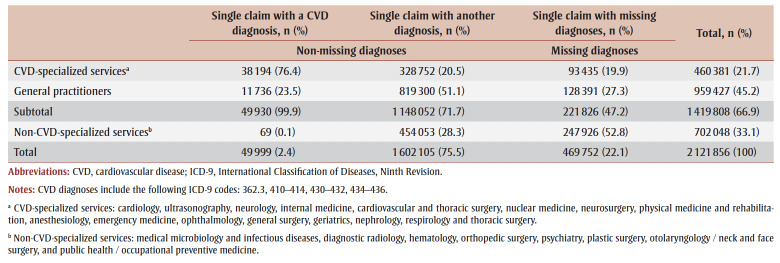

***Proportions of missing diagnoses***

**Figure 2:**
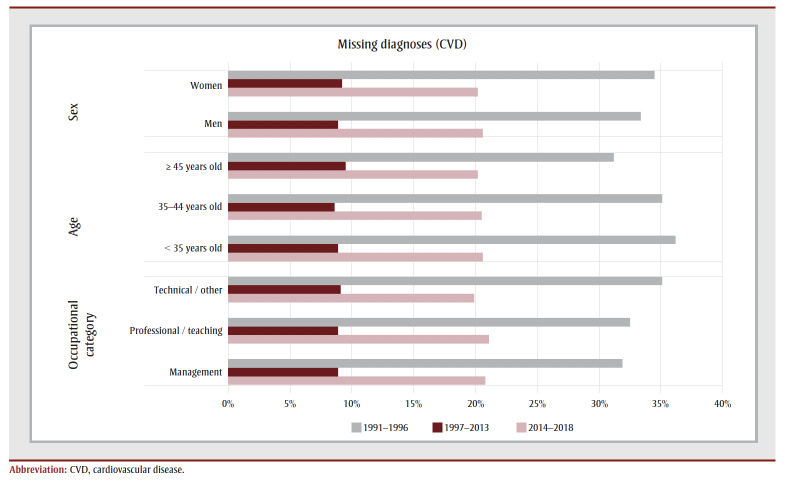
Proportion of missing diagnoses for CVD per period, by sex, age and occupational category, Quebec, Canada, 1991–2018

The proportion of missing diagnoses varied by type of services and depended on the period studied. Since the vast majority of CVD diagnoses (99.9%; 49930/49999) were associated with CVD-specialized services and general practitioners, frequency analyses were conducted based solely on claims from these services. Between 1991 and 1996, 34.0% of diagnoses were missing; between 1997 and 2013, 9.0% were missing; and between 2014 and 2018, 20.4% were missing, with an overall rate of 15.6% for the entire follow-up period. The proportions of missing diagnoses were comparable in terms of sex, age and occupational category (Figure 2).

***Number of missing CVD diagnoses***

The number of potentially missing CVD diagnoses was estimated by period and by type of services. The number of CVD diagnoses in the total number of missing diagnoses was estimated based on the claims associated with CVD-specialized services and general practitioners. For example, for a specific procedural code (09162: Primary Visit [Short-term Hospital Care, Outpatient]) performed by cardiologists between 2014 and 2018, 47.4% of the non-missing codes were CVD diagnoses and 355diagnoses were missing. Based on these proportions (i.e. 355 47.4%), there were about 168potentially missing CVD diagnoses among all the missing diagnoses for this specific combination. Adding up all the potentially missing CVD diagnoses across all types of services and for all periods equals 7389 potentially missing CVD diagnoses (Table 2), which represents a proportion of 12.9% (7389/57388) of potentially missing diagnoses among all estimated CVD diagnoses.

**Table 2:** Diagnoses and cases of known and potentially missing CVD, by study period, Quebec, Canada, 1991–2018

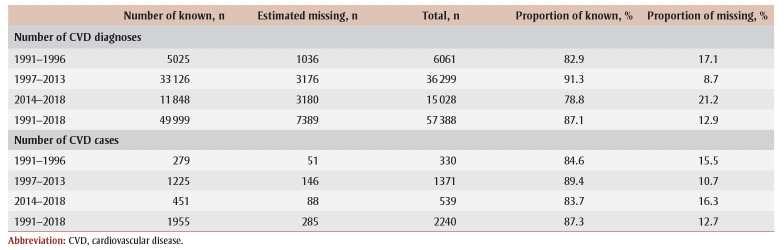

The proportion of missing diagnoses varied by the period studied: 17.1% of CVD diagnoses were potentially missing in 1991–1996, 8.7% in 1997–2013 and 21.2% in 2014–2018. A post hoc analysis conducted to determine if the proportion of missing CVD diagnoses varied between coronary heart disease and stroke determined that 12.8% of diagnoses for coronary heart disease and 13.3% of diagnoses for stroke would be missing, suggesting that there would be no difference for the two types of CVD (data not shown). 

***Number of CVD cases***

A total of 1955 CVD events were observed among the 8781 participants in our study, for an annual average of 70 cases (over 28years). The majority of CVD cases identified using the algorithms came solely from the physician claims database (i.e. 1203 non-hospitalized CVD cases out of a total of 1955 CVD cases, or 61.5%).

**Figure 3:**
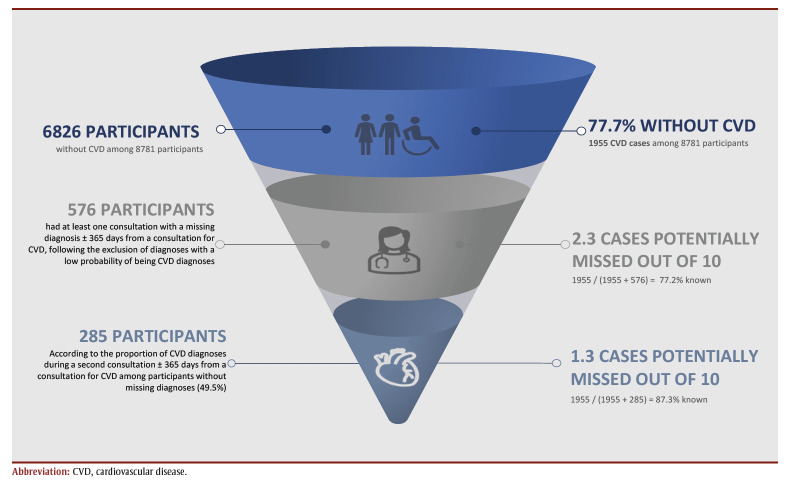
Number of potentially missing CVD cases

Of the participants without CVD according to the algorithms (n=6826/8781), 1073 non-hospitalized for CVD had only one claim for CVD and at least one other claim with a missing diagnosis within 365days before or after the claim for CVD. After excluding the missing diagnoses with a low probability of being CVD diagnoses, 576 participants had at least one other claim with a missing diagnosis within the 365-day interval that was associated with CVD-specialized services or a general practitioner. Of the participants without missing diagnoses, the proportion of CVD diagnoses in another claim, within a 365-day interval before or after a CVD claim, was 49.5%. Thus, 285 CVD cases could have been potentially missed (576 49.5%), representing an overall proportion of 12.7% of missing CVD cases (Figure 3). The proportion of missing CVD cases varied by period: 15.5% in 1991–1996, 10.7% in 1997–2013 and 16.3% in 2014–2018 period (Table 2).

## Discussion

To our knowledge, this is the first study to examine the impact of missing diagnoses on the estimated number of CVD cases in Quebec health administrative databases. The study was conducted using a large prospective cohort of 9188 males and females who were followed over 28 years. 

The results of this study suggest that the proportion of underestimated CVD diagnoses and number of CVD cases are comparable, at approximately 13%. This proportion varied depending on the period covered, with a larger underestimate at the beginning (1991–1996) and end (2014–2018) of the follow-up period.

The total proportion of missing diagnoses in the physician claims databases was 22%. This is higher than the rate observed in an earlier Quebec study, which reported that missing diagnoses varied between 7% and 12%, depending on the reference year.^8^ This difference can be explained by the 28-year follow-up of the current study, of which the more recent period (2014–2018) included a higher proportion of missing diagnoses. Similar to the Gagnon et al.^8^ study, the completeness of the diagnoses in our study varied by type of services. Gagnon et al.^8^ suggested that different compensation methods for physicians could help to explain the incompleteness of the diagnostic information in the medical services database. More specifically, fee-based compensation is more likely to affect completeness. Specialist physicians are entitled to a lump-sum payment for some medical activities, along with a supplemental fee. This mixed remuneration system could be contributing to the increase in the proportion of claims for which a diagnosis is missing.^8^ However, we also found a large number of missing diagnoses among general practitioner claims (27%), which suggests that other factors might be at play.

This study suggests that the algorithm for identifying CVD cases used for surveillance in Quebec and the rest of Canada underestimates the prevalence, incidence and burden of CVDs at the population level. Based on CCDSS administrative health data, the prevalence (standardized according to age) of ischemic heart disease was relatively stable between 2000 and 2013, while the number of people living with ischemic heart disease increased significantly, from 1.5million to 2.4million individuals.^16^ According to the INSPQ, the prevalence and incidence of CVDs decreased between 2005–2006 and 2015–2016, while the death rate remained relatively stable.^15^ For example, the incidence of ischemic heart disease decreased from 9.1 to 6.0 per 1000 population between 2005–2006 and 2015–2016.^15^ Our study findings show that the proportion of missing CVD cases varied in different periods, with the number of underestimated CVD cases seemingly higher more recently, between 2014 and 2018. The missing diagnoses in health administrative databases is thus likely to have influenced the estimation of these temporal trends.

***Strengths and limitations***

One of the strengths of this study is the long follow-up period because this allowed us to identify periods during the 28 years when the proportion of missing CVD cases was particularly high. We used INSPQ surveillance algorithms to identify CVD cases, fostering the potential to improve surveillance in Quebec.

This study has limitations. The cohort consisted only of white-collar workers, and thus is not fully representative of the Quebec population. However, several factors favour generalizing our results. First, at 52.7%, white-collar workers constitute the largest segment of the workforce in Canada.^16^ Moreover, our results suggest that the proportion of missing diagnoses does not vary according to occupational category, which is an indicator of socioeconomic status. In addition, the prevalence of exposure to known CVD risk factors, including smoking (21%) and insufficient leisure-time physical activity (21%) in this cohort is comparable to that observed in a representative sample of the Quebec population.^17^ Finally, the prevalence of CVD at recruitment, at 8%, is also comparable with the cohort observed in Canadian and American population-based surveys with a comparable age structure.^18^,^19^

It is also important to note that the algorithms used to identify CVD cases present an imperfect sensitivity (between 60% and 77%). In addition, these were validated in a different province (Ontario), using data from the Ontario Health Insurance Plan database of physician billings, which boasts virtually no missing diagnoses and near-perfect (99%) coverage.^20^ Thus, the presence of these missing diagnoses in the Quebec context is a source of underestimation in addition to that resulting from the imperfect sensitivity of the algorithms.

## Conclusion

Approximately one out of 10 CVD diagnoses is missing in the Quebec physician claims database. These missing diagnoses may have resulted in the number of CVD cases being underestimated by approximately 13% between 1991 and 2018. Underestimating CVD cases is a limitation worth considering in the context of surveillance work and epidemiological studies that use health administrative databases to identify CVD cases. Strategies aimed at improving the completeness of diagnoses in the physician claims database could be implemented and their effectiveness rigorously evaluated.

## Acknowledgements

We would like to thank all the participants in the PROspective Qubec (PROQ) Study on Work and Health. 

This work was supported the Canadian Institutes of Health Research (grant number 57750).

## Conflicts of interest

None.

## Authors’ contributions and statement

MLR: Writing – original draft, writing – review & editing

ETD: Writing – review & editing

ML: Writing – original draft, writing – review & editing

CB: Data collection, formal analysis, supervision

AM: Data curation, formal analysis

CaB: Data curation, formal analysis, interpretation

DT: Formal analysis and interpretation

XT: Supervision

All authors reviewed and approved the final draft of this manuscript.

The content and views expressed in this article are those of the authors and do not necessarily reflect those of the Government of Canada.

## References

[B01] Cardiovascular diseases [Internet]. WHO.

[B02] Leading causes of death, total population, by age group [Internet]. Statistics Canada.

[B03] (2018). Report from the Canadian Chronic Disease Surveillance System: heart disease in Canada, 2018. Government of Canada.

[B04] (2014). Economic burden of illness in Canada, 2005–2008. Government of Canada.

[B05] Gavrielov-Yusim N, Friger M (2014). Use of administrative medical databases in population-based research. J Epidemiol Community Health.

[B06] Steventon A (2013). Making the best use of administrative data: the difficulty of teasing out demand and supply. BMJ.

[B07] Blais C, Jean S, Sirois C, Rochette L, Plante C, Larocque I, et al (2014). Quebec Integrated Chronic Disease Surveillance System (QICDSS), an innovative approach. Chronic Dis Inj Can.

[B08] Gagnon R, Rochette L, Plante C (2017). Cadre de qualit des donnes du Systme intgr de surveillance des maladies chroniques du Qubec. Government of Quebec.

[B09] Wilchesky M, Tamblyn RM, Huang A (2004). Validation of diagnostic codes within medical services claims. J Clin Epidemiol.

[B10] Trudel X, Gilbert-Ouimet M, Milot A, Duchaine CS, zina M, Laurin D, et al (2018). Cohort profile: The PROspective Qubec (PROQ) Study on Work and Health. Int J Epidemiol.

[B11] Framke E, rensen JK, Andersen PK, Svane-Petersen AC, Alexanderson K, Bonde JP, et al, Heart J (2020). Contribution of income and job strain to the association between education and cardiovascular disease in 1.6 million Danish employees. Eur Heart J.

[B12] Nissen SE, Lincoff AM, Brennan D, Ray KK, Mason D, Kastelein JJ (2023). Bempedoic acid and cardiovascular outcomes in statin-intolerant patients. N Eng J Med.

[B13] Tu K, Mitiku T, Lee DS, Guo H, Tu JV (2010). Validation of physician billing and hospitalization data to identify patients with ischemic heart disease using data from the Electronic Medical Record Administrative data Linked Database (EMRALD). Can J Cardiol.

[B14] Tu K, Wang M, Young J, Green D, Ivers NM, Butt D, et al (2013). Validity of administrative data for identifying patients who have had a stroke or transient ischemic attack using EMRALD as a reference standard. Can J Cardiol.

[B15] Blais C, Rochette L (2018). Portrait de l’ensemble des maladies vasculaires au Qubec: prvalence, incidence et mortalit. Institut National de Sant Publique du Qubec.

[B16] Toronto (ON): CBRE; 2019 Nov 26 [cited 2023 Aug 24]. CBRE.

[B17] Nolin B, homme D, Godbout M (1996). L’activit physique de loisir au Qubec: une analyse en fonction des bnfices pour la sant. Kino- Qubec et Sant Qubec.

[B18] Dai S, Bancej C, Bienek A, Walsh P, Stewart P, Wielgosz A (2009). Report summary: Tracking heart disease and stroke in Canada 2009. Dai S, Bancej C, Bienek A, Walsh P, Stewart P, Wielgosz A.

[B19] Mozaffarian D, Benjamin EJ, Go AS, Arnett DK, Blaha MJ, Cushman M, et al (2015). Heart disease and stroke statistics—2015 update: a report from the American Heart Association. Circulation.

[B20] Chan BT, Schultz SE (2005). Supply and utilization of general practitioner and family physician services in Ontario. Institute for Clinical Evaluative Sciences.

